# Fine Mapping and Candidate Gene Prediction of Tuber Shape Controlling *Ro* Locus Based on Integrating Genetic and Transcriptomic Analyses in Potato

**DOI:** 10.3390/ijms23031470

**Published:** 2022-01-27

**Authors:** Guiyan Fan, Qianru Wang, Jianfei Xu, Na Chen, Wenwen Zhu, Shaoguang Duan, Xiaohui Yang, Walter S. De Jong, Yangdong Guo, Liping Jin, Guangcun Li

**Affiliations:** 1 Institute of Vegetables and Flowers, Chinese Academy of Agricultural Sciences, Key Laboratory of Biology and Genetic Improvement of Tuber and Root Crop, Ministry of Agriculture and Rural Affair, Beijing 100081, China; fanguiyan0212@163.com (G.F.); wangqianru_caas@163.com (Q.W.); xujianfei@caas.cn (J.X.); chennvnv@163.com (N.C.); god-zhuwenwen@163.com (W.Z.); duanshaoguang@caas.cn (S.D.); xiaohuiy_0601@163.com (X.Y.); 2 College of Horticulture, China Agricultural University, Beijing 100193, China; yaguo@cau.edu.cn; 3 Department of Plant Breeding, Cornell University, Ithaca, NY 14853, USA; wsd2@cornell.edu

**Keywords:** *Solanum tuberosum* L., QTL, tuber shape, candidate gene prediction

## Abstract

Tuber shape is one of the most important quality traits in potato appearance. Since poor or irregular shape results in higher costs for processing and influences the consumers’ willingness to purchase, breeding for shape uniformity and shallow eye depth is highly important. Previous studies showed that the major round tuber shape controlling locus, the *Ro* locus, is located on chromosome 10. However, fine mapping and cloning of tuber shape genes have not been reported. In this study, the analyses of tissue sectioning and transcriptome sequencing showed that the developmental differences between round and elongated tuber shapes begin as early as the hook stage of the stolon. To fine map tuber shape genes, a high-density genetic linkage map of the *Ro* region on chromosome 10 based on a diploid segregating population was constructed. The total length of the genetic linkage map was 25.8 cM and the average marker interval was 1.98 cM. Combined with phenotypic data collected from 2014 to 2017, one major quantitative trait locus (QTL) for tuber shape was identified, which explained 61.7–72.9% of the tuber shape variation. Through the results of genotyping and phenotypic investigation of recombinant individuals, *Ro* was fine mapped in a 193.43 kb interval, which contained 18 genes. Five candidate genes were preliminarily predicted based on tissue sections and transcriptome sequencing. This study provides an important basis for cloning *Ro* gene(s).

## 1. Introduction

Potato (*Solanum tuberosum* L.) is the third most important food crop in the world in terms of adaptability, yield potential, and nutritional advantages. It has a cultivation history of more than 8000 years [1], and now potato is a dual-purpose crop used for fresh food and processing. Tuber shape is one of the most important qualities in potato appearance [2] as this trait influences consumers’ willingness to purchase and trends in marketable value. For the fresh market, potatoes with regular shapes and shallow eye depth are more favored by consumers. The consumers’ preference for tuber shape may vary with regions. For processing potatoes, round tubers are typically used to make chips, while long tubers are used in the frying industry [3]. To meet the needs of the processing industries and markets, it is important to breed varieties with different tuber shapes and guarantee the stable inheritance of those shapes.

Most potato cultivars are autotetraploid (2n = 4x = 48). However, due to their highly heterozygous genome and complex genetic mechanism, little progress has been made in genetic mechanism analyses and breeding for many years [4]. Compared with diploids, tetraploids have much more complex genetic segregation ratios, and progeny analyses on the tetraploid level require a larger population [5]. The genetic mechanism of diploid potato is relatively simple, and diploid potato accounts for 70% of the existing potato germplasm resources [6]. The diploid germplasm resources with rich genetic and phenotypic variations provide an excellent opportunity for the study of various agronomic characters and disease resistance mechanisms of potato. Hence, most existing genetic research on potato tuber shape and other agronomic characters uses diploid potatoes as experimental materials [3,7,8,9,10,11,12,13].

Tuber shape varies from compressed to elongated, and the longer shapes may be straight, or kidney or sickle-shaped, or in the extreme, in a coiled form [14]. In modern varieties, to facilitate processing, shallow eyes and uniform color, round or oblong shapes have become the first choice. Through long-term artificial selection and domestication, only potatoes with uniform shapes have been preserved, which has reduced the polymorphism in potato tuber shape [15]. In the study of tuber shape, irregular shapes were usually ignored and only the length to width (LW) ratio was considered [3,16]. Tubers can be categorized into two (round, long), three (round, oval, and long), four (round, oval, long oval, very long oval), or six (long, long oval, oval, round oval, round, compressed) shapes according to LW values [12]. The estimated broad-sense heritability of shape is 0.80, indicating that tuber shape is mainly controlled by genetic factors [3].

Most previous studies on mapping of tuber shape genes were carried out in diploid potato [3,7,8,9,10,11,12,13], and some researchers believed that the tuber shape trait was controlled by a single gene [3,5,17]. A single dominant gene, termed *Ro*, was postulated by Masson [17], in which round shape was dominant over elongated, while the range of observed tuber shapes from round to oval or elongated indicates polygenic inheritance [16]. Researchers identified one major QTL locus related to tuber shape and mapped it on chromosome 10 [3,10,11,12,13], and some micro effect sites controlling potato shape were also detected in different chromosomes [11,12,13,18]. So far, no reports have been published on cloning and applications of the potato tuber shape controlling gene. To clone and apply *Ro*, we developed molecular markers and constructed a high-density genetic linkage map in the *Ro* region using a diploid segregating population, then fine mapped the *Ro* locus. Through the integration analysis of genotyping and phenotypic investigation of recombinant individuals, the *Ro* locus was fine mapped in a 193.43 kb interval containing 18 genes. The candidate genes were preliminarily predicted based on tissue sections and transcriptome sequencing. As a result, this study provides a solid foundation for cloning the *Ro* gene(s).

## 2. Results

### 2.1. Segregation of Tuber Shape in the Mapping Population

**Figure 1:**
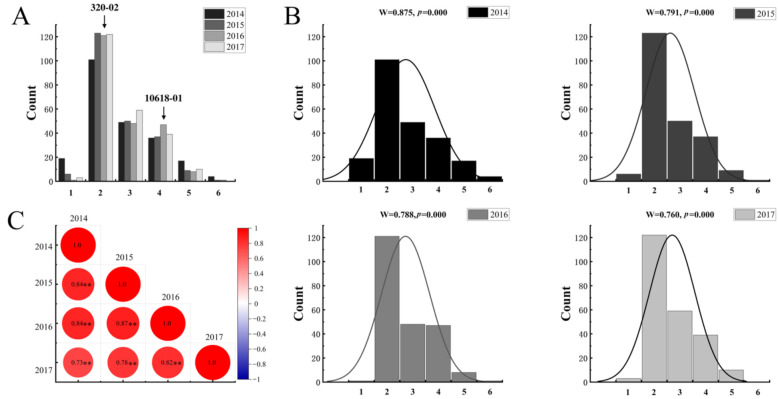
Tuber shape phenotype statistics. (**A**) Frequency distribution histogram of tuber shape (2014–2017). Different colors represent different years. The *Y*-axis indicates the tuber shape distribution, and the *X*-axis indicates the types of tuber shape: 1 = compressed, 2 = round, 3 = round-oval, 4 = oval, 5 = long-oval, and 6 = long. (**B**) Results of Shapiro–Wilk tests for the shape phenotype in different years. (**C**) Correlation analysis of tuber shape traits from 2014 to 2017; ** at the 0.01 level the correlation was significant.

There were significant differences in tuber shapes among 213 genotypes of the segregating population. The LW values of this population ranged from 0.62 to 2.62 over four years (Supplemental Table S1). According to a previous study [13], we divided the tuber shape into six types and made a frequency distribution histogram of tuber shape in the F1 population (Figure 1A,B). The two parents belonged to the second and the fourth grades, respectively. Tubers with round and round-oval shapes were more common than other shapes (Figure 1A,B). The range of LW distribution in the population was significantly larger than their parents, indicating super-parental inheritance in the mapping population. Further analysis of the Shapiro–Wilk test for the shape phenotype showed that the *p*-value is smaller than 0.05, demonstrating that the tuber shape does not conform to a normal distribution (Figure 1B), indicating that there should be a major effect-site controlling the tuber shape.

Two new potato populations, Population I and Population II, were also constructed, and their parents are the progeny of 10618-01 × 320-02. Population I was a hybrid population created by a cross between S400 (LW = 1.00) and S497 (LW = 2.13). It consisted of 64 genotypes and had a ratio of round to long tuber shape of about 1.4 (Supplemental Table S2). Population II is an inbred population of long tuber shape genotype S4 (LW = 1.84), which contained 120 genotypes, all with long tuber shapes. We randomly selected 60 genotypes for phenotyping, and the result showed that most of them had larger LW values than their parent S4 (Supplemental Table S3). This may be due to the additive effect. All the phenotypic data confirmed that the round shape was dominant over long, which was consistent with previous studies [3].

The effects of year and genotype × year interactions in this study were not significant. The broad-sense heritability of tuber shape per mean (H^2^/mean) was 0.957, showing that the phenotypic measurement error between experimental years was very small. In different years, tuber shape was generally stable, and the four-year tuber shape traits were significantly correlated (Figure 1C).

### 2.2. The Cytological Study Demonstrates the Association between the Width of Tuber and Narrowness of the Pith

**Figure 2:**
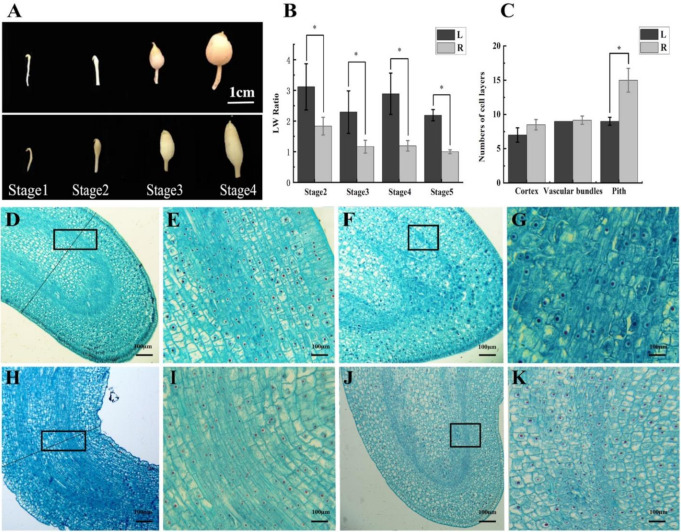
Observation of tuber phenotype and histocytology at different developmental stages. (**A**) Tuber phenotype at different developmental stages. The figure above shows the four development periods of round potato tubers, and the figure below shows the four development periods of long potato tubers. Scale bars = 1 cm (**B**) LW values of differently shaped tubers from different stages. (**C**) Numbers of cell layers in different tuber tissues in the stolon hook stage. The number of cell layers was counted at the widest position perpendicular to the elongation direction of the stolon. Error bars represent the mean ± SD, at * *p* < 0.05. (**D**,**E**) Microstructure of the round tuber at the stolon hook stage in a longitudinal section, (**E**) enlargement of the box in (**D**). (**F**,**G**) Microstructure of the round tuber at the subapical expansion stage in a longitudinal section, (**G**) enlargement of the box in (**F**). (**H**,**I**) Microstructure of the elongated tuber at the stolon hook stage in a longitudinal section, (**I**) enlargement of the box in (**H**). (**J**,**K**) Microstructure of the elongated tuber at the subapical expansion stage, (**K**) enlargement of the box in (**J**). Scale bars = 100 μm.

We divided early tuber formation into four developmental stages—stage1: stolon hook stage, stage2: subapical region expansion stage, stage3: initial tuber formation stage (0.5 cm tuber), stage4: tuber formation stage (1 cm tuber) (Figure 2A). The LW value cannot be determined in the hook stage, thus the length and width were measured in the remaining three stages, and the LW value was calculated. In addition, the mature stage of tuber development was added as stage5 and the LW value was also calculated. The LW values were significantly different between round and elongated tubers in all stages of development (Figure 2B).

Cytological differences of differently shaped tubers were identified by observing tubers from each stage. The tissues of round tubers at the hook stage were thicker than those of elongated tubers (Figure 2A). The number of cell layers at the widest position perpendicular to the elongation direction of the stolon was counted. The cell layers of the cortex, perimedullary region, and pith in the middle of young tubers were counted separately. Statistical analysis showed no significant difference in the number of cell layers in the cortex and perimedullary region, but the thickness of pith cells in round tubers was higher than that in elongated tubers (Figure 2C). Tai et al. [19] reported that the long potatoes with narrow pith were inclined to have a smaller volume of pith. Our findings were consistent with this and provided cytological support for their conclusion. The cells in the hooked phase were arranged in an orderly fashion, and there was an obvious boundary between the perimedullary bundles and the pith. The vascular tissue at the tip of round tubers had a smooth arc shape (Figure 2D,E), while the vascular bundle of elongated tubers had an arrow shape (Figure 2H,I). In the latter stages, with the development of tubers, vascular tissue became irregular, and xylem and phloem cells were dispersed in the whole perimedullary regions (Figure 2F,G,J,K). There was little difference in the cell morphology between long and round potato shapes.

### 2.3. Patterns of Differential Gene Expression Reveal That Tuber Shape Determination Occurred in the Early Stage of Tuber Development

**Figure 3:**
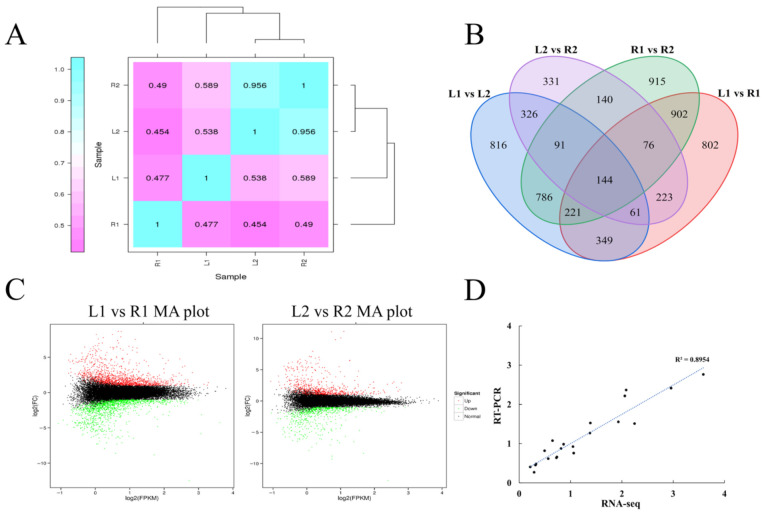
RNA-seq analysis. (**A**) Heat map of expression quantity correlation of each sample. (**B**) Venn plot for DEGs per comparison. Non-overlapping numbers represent the number of genes unique to each pair. Overlapping numbers represent the number of mutual genes between groups. (**C**) Ma plot at different development stages of tubers, each point represents a gene, the abscissa indicates the log2(FPKM); the ordinate indicates log2(fold change). The red dots indicate up-regulated expression of the genes, the green dots indicate down-regulated expression of the genes, and the black dots indicate no statistically significant difference in expression of the genes. (**D**) Correlation analysis of data from RNA-Seq and qRT-PCR.

According to histocytological observation, the stolon in the hook stage and the subapical region expansion stage were selected to construct the mixed pool for transcriptome sequencing. Each pool contained 22 genotypes, and tuber RNA was mixed in equal amounts to create the sequencing library. R1 and R2 represent the round tuber hook stage and the subapical region expansion stage, respectively. L1 and L2 represent the elongated tuber hook stage and the subapical region expansion stage, respectively. After sequencing quality control, a total of 29.83 Gb clean data was obtained, and the percentage of Q30 bases of all products was not less than 94.06%. The comparison efficiency between the reads of each sample and the reference genome was 81.39–86.12%. Pearson correlation coefficient (R^2^) analysis between the gene expression of the four samples was performed. The R^2^ between L2 and R2 was 0.956 (Figure 3A), demonstrating little difference in gene expression at the subapical region expansion stage of tuber development. In contrast, the R^2^ between L1 and R1 was 0.477, which is lower than L2 and R2, illustrating the big difference of the gene expression in the hook stage. This result confirmed that tuber shape determination occurred in the early stage of tuber development.

Significant differentially expressed genes (DEGs) were chosen using threshold values (absolute value of log2(fold change) > 1 and false discovery rate (FDR) < 0.01). There were 2778 DEGs in the R1 and L1 pools, of which 1353 genes were up-regulated and 1425 genes were down-regulated. In the subapical expansion stage, there were 1392 DEGs in the R2 and L2 pools, of which 728 genes were up-regulated and 664 genes were down-regulated (Figure 3B,C). The number of DEGs was higher in the early stage of development, indicating that the tuber shape determination period may have occurred early in development.

To confirm the reliability of the RNA-seq data, 20 DEGs were randomly selected to perform qRT-PCR (Supplemental Table S4). The results showed that the transcript expression levels of qRT-PCR and RNA-seq were highly correlated (R^2^ = 0.895) (Figure 3D), demonstrating that the RNA-seq results were reliable.

### 2.4. Functional Classification of DEGs Showed That Tuber Shape May Be Related to Auxin and Cell Wall Formation Pathways

The DEGs were functionally annotated to analyze their gene ontologies (GO) to assess their functional enrichment. The top enriched DEGs of the two stages had some similar biological processes: response to auxin (GO: 0009733), cell wall biogenesis (GO: 0042546), regulation of organ growth (GO: 0046620), and cell redox homeostasis (GO: 0045454). Most of them were biological processes related to auxin response and cell wall development.

**Figure 4:**
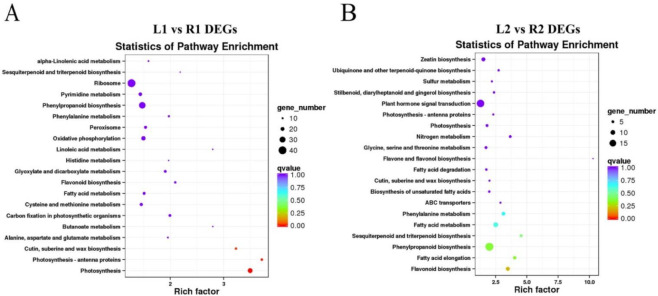
The top 20 enriched KEGG pathways of DEGs. (**A**) KEGG enrichment analysis of DEGs between L1 and R1, (**B**) KEGG enrichment analysis of DEGs between L2 and R2. The *x*-axis represents the rich factor and the *y*-axis represents the pathway name. The size of a circle represents the DEG number.

In organisms, different gene products coordinate with each other to perform biological functions. The annotation analysis of the pathway is helpful to further interpret the functions of DEGs. We performed Kyoto Encyclopedia of Genes and Genomes (KEGG) enrichment analysis to determine the biological pathways involved in the DEGs. KEGG enrichment analysis showed that the major pathways involving DEGs were as follows: “flavone and flavonol biosynthesis”, “photosynthesis”, “phenylpropanoid biosynthesis”, and “cutin, suberine, and wax biosynthesis” (Figure 4). Flavonoids, a class of secondary plant metabolic compounds, have been suggested to be auxin transport inhibitors. The pathway of “cutin, suberine, and wax biosynthesis” is related to cell wall synthesis. The genes which related to auxin response and cell wall development were significantly enriched in the top 20 pathways, suggesting that these pathways may affect tuber shape.

### 2.5. Fine Mapping of Ro in the Defined Genomic Region

**Figure 5:**
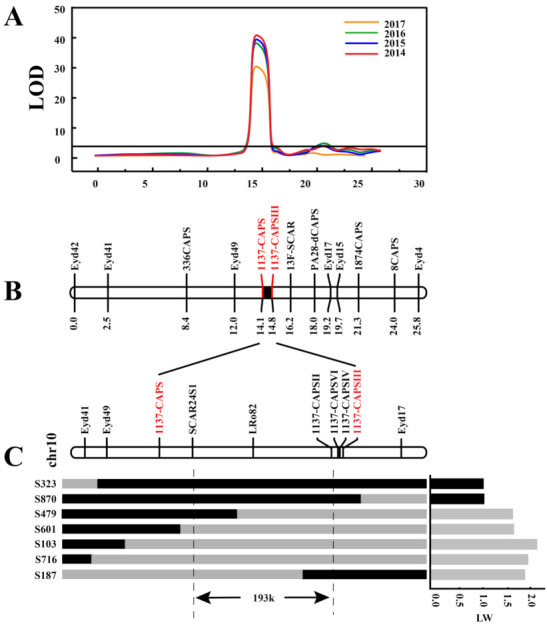
Fine mapping of *Ro*. (**A**) Analysis of QTLs for *Ro* on chromosome 10 for 2014–2017. (**B**) Genetic linkage map of *Ro* locus. (**C**) Fine mapping of *Ro* by recombinant screening. Black bars indicate the heterozygous genotype of 320-02, and gray bars indicate the homozygous recessive genotype.

The major QTL on chromosome 10 plays the most important role in tuber shape, but fine mapping of *Ro* needs to be performed. Primers were developed at 1–3 Mb intervals in the candidate region of *Ro* on chromosome 10 based on our previous BSA sequence data [20] (Supplemental Table S5). Using the molecular markers developed in this and a previous study, a genetic linkage map on chromosome 10 was constructed (Figure 5). The linkage map contained 13 markers, and the total length was 25.8 cM with an average marker interval of 1.98 cM. Combined with phenotypic data from four years, a QTL for tuber shape was detected on chromosome 10, and the LOD value reached 31.5–42.0 (Figure 5A), which explained 61.7–72.9% of the variation (Table 1). The *Ro* candidate loci were located between the molecular markers, 1137-CAPS and 1137CAPSIII, with a physical distance of 224.59 kb (Figure 5B).

For fine mapping of the *Ro* locus, more polymorphic markers such as LRo82 were developed in the targeted region for genotyping to narrow down the candidate genomic region. These polymorphic markers were subsequently utilized for genotyping individuals of the 10618-01 × 320-02 population. A total of 7 recombinant plants for the determinate type were identified for further genotyping. Based on the genotyping and phenotyping results of these 7 recombinant plants, the *Ro* locus was finally located in a genomic region flanked by markers SCAR24S1 (chr10:49534856) and 1137CAPSII (chr10:49728413), with an interval size of 193.6 kb (Figure 5C).

### 2.6. Five Differentially Expressed Genes Are Pinpointed as Candidate Genes Involved in Tuber Shape Formation

**Figure 6:**
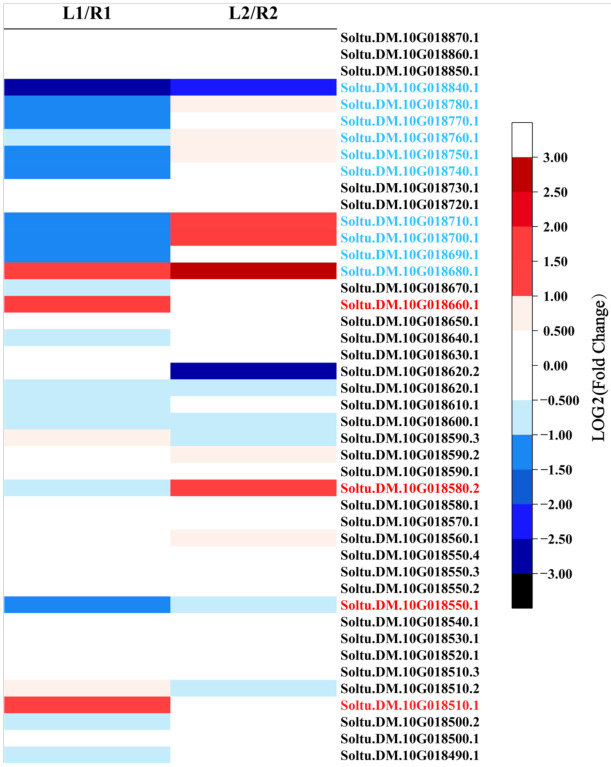
The expression fold change of candidate and flanking genes. The left side represents the change of gene expression in the hook stage, the right side represents the change of gene expression in the subapical region expansion stage. The red font indicates the DEGs in the candidate interval, and the blue font indicates the DEGs in the flanking interval. Each square represents a single gene, and each gene occupies equivalent positions in each set.

Based on the annotation information of the reference genome (DM v6.1: http://spuddb.uga.edu, accessed on 21 January 2022), 18 genes in this interval were predicted (Table 2). Combined with RNA-seq data, five genes were shown to be differentially expressed and could be considered as candidate genes involved in tuber shape formation: *Soltu.DM.10G018510* (nuclear shuttle interacting gene), *Soltu.DM.10G018550* (aminophospholipid ATPase), *Soltu.DM.10G018580* (lung seven transmembrane receptor family protein), *Soltu.DM.10G018620* (*HSI2-like*), and *Soltu.DM.10G018660* (lipid transfer protein) (Figure 6).

*Soltu.DM.10G018620*(*HSI2-like*) is a class of transcription inhibitors, which contains the plant specific B3 domain. According to structural characteristics and functions, the B3 domain family can be divided into five subfamilies: auxin response factor (*ARF*), abscisic acid-insensitive 3 (*ABI3*), high level expression of sugar inducible (*HIS*), related to ABI3/VP1 (*Rav*), and reproductive meristem (*REM*). These gene families function directly in regulating plant growth and development, organ morphogenesis, flower bud differentiation, and responding to a variety of stresses [21,22]. *Soltu.DM.10G018660*(*nsLTP*) is a small molecular protein, which has been reported as a cell wall-loosening protein to facilitate wall extension [23]. The functions of these candidate genes are consistent with the biological pathway of differential gene enrichment in RNA sequencing, which is the object of our further research.

## 3. Discussion

### 3.1. Characterization of the Mapping Population

In the past, taxonomy for tuber shape was either based on the appearance of tuber shape or the LW value [7,24,25]. For the appearance-based taxonomy, the general tuber shape was scored by visual examination according to the morphological descriptors. Tuber shape was divided into two, three, four, five, or even eight categories. The International Potato Center (CIP) currently divides shape categories into 17 classes, including compressed, round, ovoid, obovoid, elliptic, oblong, long-oblong, elongated and so on [24]. These 17 different tuber shapes are recognized as formal morphological descriptors, but it is unknown whether all this variation can be explained by different alleles at the *Ro* locus [12]. The second category uses the LW as the phenotypic value for tuber shape [3,25]. For the LW value-based taxonomy, the irregular shapes are usually ignored and only the length to width ratio is considered [3,16]. We combined the LW value with phenotypic classification. The LW values of the segregating population used in this study ranged from 0.62 to 2.62. The phenotype changed from compressed to long, and there were no other shapes such as bending or claw shapes. A continuous distribution of phenotypes was evident for the ratio of tuber length to width and a strong QTL for tuber shape trait was identified on chromosome 10 in our study.

In previous studies, phenotypic evaluation was based on 2- or 3-year experiments for mapping and analysis [9,11,13]. To get more accurate results, our study investigated the tuber shape of the same population planted in the same area for four years, and the results showed that inheritance of the tuber shape trait was stable. Through our four-year evaluation, we found that extreme shapes decreased over time, which might be due to improper field management causing the plants to degenerate. Another reason could be that there were too many heterozygous loci in the parental genotypes and large non-additive gene effects among genes. The non-additive gene effects gradually lessened during planting, which led to the degradation of traits.

### 3.2. Mapping of the Ro Gene

Cultivated potato is an autotetraploid species with a highly heterozygous genome and complicated inheritance. Although a series of tools for linkage mapping and QTL analyses of tetraploid potato has been developed [26,27], due to the complexity of tetraploids, most studies of genetic analysis, mapping populations, and genetic map construction are still based on diploid potatoes. Based on the linkage map, markers closely linked to genes that controlled target traits could be easily found and used for gene mapping and cloning. For QTL mapping, in order to find relevant QTLs, the average interval of markers should be below 10 cM, and the average interval of target region markers should be below 1 cM for cloning. In this study, a genetic linkage map of the *Ro* region on chromosome 10 with an average marker interval of 1.98 cM was constructed, which met the needs of QTL mapping. Researchers have determined that the main gene controlling potato shape is located on chromosome 10 [3,9,11,12], but fine mapping of *Ro* needs to be performed. In this study, through genotype–phenotype joint analysis of the recombinant plants, the *Ro* locus was located in a genomic region flanked by markers SCAR24S1 and 1137CAPSII, with an interval size of 193.6 kb. Based on the annotation information of DM v6.1, 18 genes in this interval were predicted, which provides excellent help for later candidate gene screening.

### 3.3. Candidate Gene Prediction

Compared with conventional fine-mapping processes, integrating QTL mapping and RNA-sequencing is more conducive to rapidly identifying candidate genes related to target traits within major QTLs, thus providing a rapid approach for mapping target genes. Patterns of differential gene expression revealed the tuber shape determination occurred in the early stage of tuber development. RNA sequencing data showed five DEGs in this candidate interval may be involved in tuber shape formation: *Soltu.DM.10G018510* (nuclear shuttle interacting gene), *Soltu.DM.10G018550* (aminophospholipid ATPase), *Soltu.DM.10G018580* (lung seven transmembrane receptor family protein), *Soltu.DM.10G018620* (*HSI2-like*), and *Soltu.DM.10G018660* (lipid transfer protein) (Figure 6). In order to further confirm the results, we verified the candidate genes by qRT-PCR. qRT-PCR results showed that the remaining genes were consistent with RNA-seq results, and there were significant differences between round tuber and long tuber.

Of these candidate genes, *HSI2-like* belongs to the plant-specific B3 DNA binding domain superfamily. The B3 family can be divided into five subfamilies: *ARF*, *ABI3*, *HIS*, *Rav,* and *REM* [28]. B3 transcription factors have diverse functions in plant growth and development. The *ARF* family functions in auxin response can be detected in a variety of tissues and are essential in regulating plant growth and development. Class C *ARFs* inhibit cell differentiation and growth in many tissues, including roots, leaves, and flowers. The *AtARF5* gene acts in controlling the development of microtubules and hypocotyls [29]. The biological function of this gene family is related to cell wall development and the auxin response, which corresponded to the transcriptome results, and the expression of this gene is different in tubers with different shapes, so it may be related to potato shape.

The last gene in this candidate interval was a gene cluster containing 18 genes on the flank of the candidate interval. This cluster is a large transporter family named non-specific lipid transfer protein (nsLTP). There are 83 *nsLTP* genes in potatoes, which are mainly expressed in younger tissues [30]. Many *nsLTP* genes have a high degree of tissue specificity and are expressed at key stages of the potato life cycle. A previous study showed that *nsLTP* was specifically expressed in the tuber vascular bundle [31]. In addition, *nsLTP* has a regulatory function in some hormone responses and signaling, such as ethylene, methyl jasmonate, and abscisic acid (ABA) [32]. Transcriptome results showed that many *nsLTP* had big differences between round and elongated tubers in early developmental periods. These genes may thus have a role in regulating tuber shape.

## 4. Materials and Methods

### 4.1. Plant Material

The tuber shape segregating population and its parents, provided by Cornell University (Ithaca, NY, USA), were diploid genotypes, and their progeny were propagated into replicated experiments with potato tubers. The female parent, 10618-01, had a long tuber shape (LW = 1.72), purple skin and flesh, and the *Ro* site was recessive homozygous; the male parent, 320-02, had a round tuber shape (LW = 0.92), yellow skin and flesh, and the *Ro* site was heterozygous [7]. The progeny consisted of 213 genotypes. From 2014 to 2017, potatoes were planted in Hebei province in the net-house of Zhangbei County Experimental Base in Zhangjiakou City (41°15′ N, 114°7′ E, during potato growing in the field from early May to late September, the minimum and maximum temperatures ranged from 10.05 °C to 21.66 °C, and the day length duration was 8.54 h). In addition, two other populations, Population I and Population II, were used in this study, and their parents are the progeny of 10618-01 × 320-02. Population I is a hybrid population from the cross between S400 (LW = 1.00) and S497 (LW = 2.13); Population II is an inbred population of long tuber shape genotype S4 (LW = 1.84). The parents and all the genotypes of their segregating population were randomly planted in the field, and each genotype had four plants, with each plant considered a replicate. Potato tubers were harvested once potato plants reached physiological maturity in late September.

### 4.2. Potato Shape Evaluation and Analysis

Three replicate plants of each genotype with uniform growth were selected and their fully developed tubers were measured with vernier calipers. The length was the maximum distance of the tuber parallel to the growth direction of the stolon, and the width was the maximum distance of the growth direction for the vertical stolon. The LW of three tubers was calculated for each genotype.

Tuber shape was determined according to the Hara–Skrzypiec research [13], which described six tuber shapes: tubers with LW ≤ 0.8 are compressed; 0.8 < LW ≤ 1.2 are round; 1.2 < LW ≤ 1.6 are round-oval; 1.6 < LW ≤ 2 are oval; 2 < LW ≤ 2.4 are long-oval, and LW > 2.4 are long. The Shapiro–Wilk test was calculated for data from 2014–2017 using SPSS V. 19.0 (SPSS, Inc., Chicago, IL, USA). Broad-sense heritability was estimated from the analysis of variance according to the formula of Hara–Skrzypiec [13], H^2^ = σ^2^g/(σ^2^g + σ^2^ge + σ^2^e); σ^2^g = (M1 − M2)/L; σ^2^ge = M2 − σ^2^e; where M1 = mean sum of squares effect of genotype, M2 = mean sum of squares effect of genotype × year, and L = number of years.

### 4.3. Tissue and Cell Observation

In order to study the differences in cell structure between round and elongated tubers in tuber initiation and development, three round (0.91 < LW < 1.1) and three elongated (1.93 < LW < 2.39) potato tubers were selected for slice observation. (1) Four developmental stages: stolon hook stage, subapical region expansion stage, initial tuber formation stage (0.5 cm tuber), and tuber formation stage (1 cm tuber) were picked to prepare paraffin sections. (2) Fresh tubers were taken from different development stages and fixed in a formaldehyde–acetic acid–alcohol (FAA) solution (V50% ethanol: V formaldehyde: V glacial acetic acid = 8:1:1) for 24 h. (3) For dehydration, fixed materials were picked up with tweezers and put into ethanol with different concentration gradients. (4) Samples were dipped in wax after they were made transparent by xylene. (5) Materials were embedded then sliced with a Leica EM UC6 ultramicrotome (Leica Microsystems GmbH, Wetzlar, Germany), and the slicing thickness was about 4 μm. (6) After dewaxing, samples were dyed with safranine and solid green. (7) The sections were observed and imaged using an OLYMPUS CX31 (Tokyo, Japan) microscopy [33,34]. All tubers were sectioned lengthwise, and only the near median sections were used for observation. At the widest position perpendicular to the elongation direction of the stolon, the number of cell layers in the cortex, perimedullary region, and pith were counted separately. Each potato shape had three biological repetitions. After counting the number of cell layers in each part, single-point ANOVA in Excel was used to test whether the difference was significant.

### 4.4. RNA-seq Analysis

Total RNAs were extracted from the tubers. RNA concentration was measured using a NanoDrop 2000 (Thermo, Waltham, MA, USA). RNA integrity was assessed using the RNA Nano 6000 Assay Kit of the Agilent Bioanalyzer 2100 system (Agilent Technologies, CA, USA). The library preparations were sequenced on an Illumina platform and paired-end reads were generated. RNA-seq data were aligned to the reference genome (DM v6.1) by Hisat2 tools [35] and expression levels were quantified using StringTie V1.3.6 [36]. Ebseq was used for differential analysis. DEGs were chosen using threshold values (absolute value of log2(fold change) > 1 and FDR < 0.01).

To validate the results of the RNA-seq data, 20 DEGs were randomly selected to perform qRT-PCR. The gene-specific primers (Supplemental Table S4) of the selected genes, ef1a as the internal reference gene, were designed using NCBI online Primer-BLAST (https://www.ncbi.nlm.nih.gov/tools/primer-blast/index.cgi?LINK_LOC=BlastHom, accessed on 20 January 2022). The RNA samples were identical to the samples used for RNA-seq analysis. HiScript^®^ III 1st Strand cDNA Synthesis Kit (Vazyme, Nanjing, China) was used for reverse transcription. Total RNA (1 μg) was used and after cDNA synthesis, it was diluted five times for later experiments. The PCR reaction system was 20 μL containing 10 μL of Taq Pro Universal SYBR qPCR Master Mix, 0.4 μL each of forward and reverse primers, 7.2 μL of double distilled water, and 2 μL of cDNA sample. The expression level of each gene was calculated using the 2^(−^^△^^△CT)^ method [37]. The correlation coefficient of RNA-seq and qRT-PCR was calculated to verify the reliability.

### 4.5. Molecular Marker Development

In our previous studies, two separate DNA hybrid pools were constructed using extremely long potato shapes and round potato shapes [20]. Each sample was sequenced at 30× coverage of the assembled genome by the Illumina HiSeqTM 2500 platform. The clear reads from the two parental and two progeny pools were mapped to the DM v6.1 reference genome using BWA (v0.7.13). GATK software [38] was used to detect multiple samples of single nucleotide polymorphisms (SNPs) and insertions or deletions (InDel). The SNP index was calculated for both the round and the long bulk samples by measuring the proportion of reads containing SNPs that were identical to those in the round parent (320-02). The ΔSNP index was calculated as (SNP index of the round pool) – (SNP index of the long pool). The sites with ΔSNP index absolute value greater than 0.75 were screened to develop markers. SNP and InDel markers between the two pools at 1–3 Mb intervals in the candidate region of *Ro* on chromosome 10 were selected to develop tuber shape linkage markers [20]. All primers were synthesized by Sangon Biotechnology Service Co., Ltd. (Shanghai, China). The primers were first screened using the parents and the polymorphic primers between the parents were further used to verify the primer polymorphisms in the F1 population (213 genotypes).

### 4.6. Genetic Linkage Map Construction and QTL Mapping

The designed primers were screened for polymorphism using the DNA of the parents and 213 genotypes in the segregating population. The genetic linkage map of tuber shape was constructed with joinmap4.0 software [39], combined with the phenotypic data, and QTL analysis was done using MQM mapping with software MapQTL^®^6 [40]. Detection of QTLs was conducted using LOD threshold > 3 after 1000 permutation tests, and a chromosome-wide node was used to judge whether the QTL peak was significant.

### 4.7. Identification of the Candidate Genes

According to the results of QTL mapping, border markers were used to screen the tuber shape segregating population to find individual plants with the recombinant exchange. To fine map the tuber shape location, new molecular markers were developed in the target range to detect individual recombinant plants. According to the phenotypic data and marker typing results, the candidate interval was determined. Annotation information of the potato reference genome DM v6.1 was used to conduct functional analyses of all genes within the QTL interval to assist in the selection of candidate genes.

## 5. Conclusions

The cytological study demonstrates the association between the width of the tuber and the narrowness of the pith in the hook stage. The expression pattern of tuber development genes was investigated by transcriptome analysis, and the results revealed that the tuber shape determination occurred in the early stage of tuber development. A major locus that controlled tuber shape on potato chromosome 10 was identified based on four years of phenotypic and genotype association analyses. Further, through the marker typing and phenotypic investigation of recombinant individuals, the *Ro* locus was fine mapped in a 193.56 kb interval containing 18 genes. Five DEGs were predicted as candidate genes involved in tuber shape formation. This study provides an important basis for the cloning of *Ro* gene(s), and markers linked to tuber shape could be used for marker assistant breeding.

## Figures and Tables

**Table 1:** QTLs for tuber shape detected in the segregating population.

Year	QTL	LOD	PVE (%)	Additive Effect	Marker Interval	Position
2014	1137CAPS	42.00	72.9%	0.433438	1137CAPS–137CAPSIII	chr10: 49510442–49735039
2015	1137CAPS	40.29	71.4%	0.364612	1137CAPS–1137CAPSIII	chr10: 49510442–49735039
2016	1137CAPS	39.87	71.3%	0.357161	1137CAPS–1137CAPSIII	chr10: 49510442–49735039
2017	1137CAPS	31.51	61.7%	0.304663	1137CAPS–1137CAPSIII	chr10: 49510442–49735039

**Table 2:** Candidate genes in *Ro* mapping interval.

Name	Description
*Soltu.DM.10G018490*	Leucine-rich repeat protein kinase family protein
*Soltu.DM.10G018500*	Leucine-rich repeat protein kinase family protein
*Soltu.DM.10G018510*	Nuclear shuttle interacting
*Soltu.DM.10G018520*	Glycosyl hydrolase superfamily protein
*Soltu.DM.10G018530*	Serine/threonine protein kinase
*Soltu.DM.10G018540*	Hypothetical protein
*Soltu.DM.10G018550*	Aminophospholipid ATPase
*Soltu.DM.10G018560*	WRKY DNA-binding protein
*Soltu.DM.10G018570*	Protein of unknown function, DUF599
*Soltu.DM.10G018580* *Soltu.DM.10G018590*	Lung seven transmembrane receptor family protein Vacuolar sorting receptor homolog
*Soltu.DM.10G018600*	Polynucleotidyl transferase, ribonuclease H-like superfamily protein
*Soltu.DM.10G018610*	Elongation factor P (EF-P) family protein
*Soltu.DM.10G018620*	HSI2-like
*Soltu.DM.10G018630* *Soltu.DM.10G018640*	Hypothetical protein HSI2-like
*Soltu.DM.10G018650*	Protein kinase superfamily protein
*Soltu.DM.10G018660*	Non-specific lipid transfer protein

## Data Availability

Not applicable.

## Supplementary Materials

The following are available online at https://www.mdpi.com/article/10.3390/ijms23031470/s1.
