# Supplementary material for: Fine Mapping and Candidate Gene Prediction of Tuber Shape Controlling Ro Locus Based on Integrating Genetic and Transcriptomic Analyses in Potato
Source: Int J Mol Sci. 2022 Jan 27;23(3):1470. doi: 10.3390/ijms23031470 (PMC8836246; doi:10.3390/ijms23031470)
Supplement: Supplementary file 1 [file ijms-23-01470-s001.zip › ijms-1551071-supplementary.pdf]

Supplemental Table S1：The mean and ranges of progeny length/width (LW) of the parental and F1 population from 2014 to 2017.

Year	Male parent	Female parent	Population mean	Range of individual values
2014	0.91	2.04	1.27	0.62-2.62
2015	0.99	1.94	1.23	0.72-2.42
2016	1	1.95	1.28	0.72-2.58
2017	1	1.84	1.25	0.72-2.36

Supplemental Table S2：LW values of population Ⅰ

Materials number	LW	Description
S400（male parent）	1.01	round
S497（female parent）	1.77	oval
S400497-60	0.75	compressed
S400497-64	0.78	compressed
S400497-20	0.91	round
S400497-89	0.94	round
S400497-83	1.00	round
S400497-66	1.01	round
S400497-45	1.02	round
S400497-90	1.02	round
S400497-47	1.05	round
S400497-56	1.06	round
S400497-30	1.07	round
S400497-50	1.08	round
S400497-1	1.09	round
S400497-73	1.10	round
S400497-32	1.11	round
S400497-7	1.13	round
S400497-49	1.14	round
S400497-34	1.15	round
S400497-55	1.17	round
S400497-68	1.18	round
S400497-82	1.20	round
S400497-11	1.25	round-oval
S400497-19	1.28	round-oval
S400497-63	1.31	round-oval
S400497-33	1.32	round-oval
S400497-84	1.32	round-oval
S400497-24	1.34	round-oval
S400497-74	1.36	round-oval
S400497-67	1.37	round-oval
S400497-61	1.42	round-oval
S400497-9	1.45	round-oval
S400497-23	1.48	round-oval
S400497-59	1.51	round-oval
S400497-72	1.55	round-oval

Materials number	LW	Description
S400497-26	1.57	round-oval
S400497-85	1.57	round-oval
S400497-76	1.58	round-oval
S400497-46	1.61	oval
S400497-88	1.64	oval
S400497-22	1.67	oval
S400497-3	1.67	oval
S400497-41	1.74	oval
S400497-97	1.74	oval
S400497-59	1.75	oval
S400497-2	1.78	oval
S400497-57	1.79	oval
S400497-71	1.80	oval
S400497-78	1.80	oval
S400497-79	1.85	oval
S400497-4	1.88	oval
S400497-48	1.96	oval
S400497-13	1.97	oval
S400497-25	2.01	long-oval
S400497-43	2.02	long-oval
S400497-29	2.07	long-oval
S400497-92	2.24	long-oval
S400497-81	2.31	long-oval
S400497-70	2.34	long-oval
S400497-58	2.37	long-oval
S400497-51	2.39	long-oval
S400497-28	2.41	long
S400497-86	2.45	long
S400497-75	2.66	long
S400497-21	2.89	long

Supplemental Table S3: LW values of population II

Materials number	LW	Description
S4 (inbred parent)	1.79	oval
S4-112	1.67	oval
S4-66	1.70	oval
S4-149	1.73	oval
S4-21	1.76	oval
S4-4	1.78	oval
S4-110	1.83	oval
S4-147	1.85	oval
S4-48	1.87	oval
S4-155	1.89	oval
S4-76	1.90	oval
S4-108	1.90	oval
S4-7	1.92	oval

Materials number	LW	Description
S4-1	1.93	oval
S4-191	1.93	oval
S4-107	1.93	oval
S4-168	1.96	oval
S4-39	1.97	oval
S4-63	1.99	oval
S4-12	2.00	oval
S4-167	2.00	oval
S4-148	2.02	long-oval
S4-27	2.03	long-oval
S4-178	2.05	long-oval
S4-171	2.08	long-oval
S4-109	2.09	long-oval
S4-195	2.09	long-oval
S4-182	2.09	long-oval
S4-115	2.10	long-oval
S4-187	2.10	long-oval
S4-75	2.14	long-oval
S4-119	2.14	long-oval
S4-16	2.14	long-oval
S4-196	2.15	long-oval
S4-8	2.15	long-oval
S4-98	2.16	long-oval
S4-77	2.16	long-oval
S4-26	2.17	long-oval
S4-9	2.21	long-oval
S4-170	2.24	long-oval
S4-142	2.28	long-oval
S4-154	2.30	long-oval
S4-17	2.30	long-oval
S4-133	2.30	long-oval
S4-6	2.32	long-oval
S4-73	2.35	long-oval
S4-176	2.36	long-oval
S4-161	2.38	long-oval
S4-192	2.39	long-oval
S4-151	2.39	long-oval
S4-163	2.43	long
S4-129	2.46	long
S4-69	2.47	long
S4-70	2.51	long
S4-136	2.55	long
S4-55	2.56	long
S4-183	2.63	long
S4-68	2.69	long
S4-101	2.77	long
S4-162	2.77	long

Materials number	LW	Description
S4-127	2.78	long
S4-88	2.85	long
S4-3	2.89	long
S4-93	2.89	long
S4-175	3.03	long
S4-57	3.05	long
S4-121	3.16	long
S4-125	3.17	long
S4-116	3.21	long
S4-47	3.21	long
S4-152	3.29	long
S4-139	3.32	long
S4-96	3.42	long
S4-53	3.82	long
S4-145	3.86	long
S4-124	3.98	long
S4-89	4.03	long
S4-156	4.40	long
S4-64	4.57	long
S4-80	5.08	long
S4-84	6.28	long

Supplemental Table S4: Primers for qRT-PCR

Number	Gene ID	Primer Name	Primer
1	Soltu.DM.12G026390.1	Glucose-1-F	ACTTCTCGTACTCCGAAACGG
		Glucose-1-R	AACCAATCCCCTTAGCCGTG
2	Soltu.DM.07G027830.1	Glucose-2-F	TCAGTGGCGTACAACACCAT
		Glucose-2-R	GTGCTGCTCCGTGGTATTCA
3	Soltu.DM.07G003310.1	Glucose-3-F	CATCACGGAGGCGATAAGGG
		Glucose-3-R	GACTTTCGAGTCCGGCTTCA
4	Soltu.DM.12G000940.1	Glucose-4-F	TCTTGGTCCTTCCATCGGGT
		Glucose-4-R	CAACGCCTGTCTCATCTGCT
5	Soltu.DM.04G027260.1	ABA-1-F	ACATCACCGTTTGTTCCACC
		ABA-1-R	TTTGCCTTGTGCTTCTCATGC
6	Soltu.DM.06G010300.2	ABA-2-F	CGCCGATTCGATAACCCACA
		ABA-2-R	CGGAAGTCCCGATACAACCA
7	Soltu.DM.02G017330.1	IAA-1-F	TGGTCCCAGCCCTGATAAAG
		IAA-1-R	TTGTCGGCATCGACAGAGAC
8	Soltu.DM.03G001970.1	IAA-2-F	GGAGCAAGCAAGAGCTACCA
		IAA-2-R	GGATTGAACACGCTCCTCCA
9	Soltu.DM.09G025690.1	IAA-3-F	TGAGCTGAGGTTAGGTTTGCC
		IAA-3-R	GGCAACAGGTGGGGTTTTGA
10	Soltu.DM.01G032750.1	GA--1-F	GAGGCATGTCGAAAGCATGG
		GA-1-R	ACAAGGGGCTTCCCAAAGAA
11	Soltu.DM.02G023870.1	GA--2-F	GAGGTGCAGCAAATCGTCTC
		GA-2-R	AGCAAGGGCATGCTTCGTAA

12	Soltu.DM.02G013470.1	GA--3-F	AGTCCCACCTGATCCCTACTC
		GA-3-R	TGGCCCTCCAAAGTAAACCA
13	Soltu.DM.10G018490.1	490qPCR-F	AATGGAACTTTGCGAAACTGGC
		490qPCR-R	TTGGTGGATGAGCTTCTGTATG
14	Soltu.DM.10G018500.1	500qPCR-F	GACGGAGGTGAAGAGTATGGAG
		500qPCR-R	ATGTGGTGGAAAGTCTGAAATG
15	Soltu.DM.10G018530.1	530-RLK-qPCR-F	GCCATGCCATGTTGACTGAT
		530-RLK-qPCR-R	AAAGGAGGCTTGCCCGTGAG
16	Soltu.DM.10G018560.1	560-WRKY-qPCR-F	TCTCCGAATTTATTCTACCC
		560-WRKY-qPCR-R	CTGATGACAATGCTGCTCTA
17	Soltu.DM.10G018550.1	550-AAase-qPCR-F	AAGCCACCACGACATACAAA
		550-AAase-qPCR-R	TTGCCACTCATCCAGTTCTTC
18	Soltu.DM.10G018580.1	580-lung-qPCR-F	TTTCAATGGGTTATGGTGTC
		581-lung-qPCR-R	TTTCAATGGGTTATGGTGTC
19	Soltu.DM.10G018610.1	610-EF-P-qPCR-F	CAGGGAACAGCGTTGAGAAA
		610-EF-P-qPCR-R	TGAAGACATACTGGGCACCA
20	Soltu.DM.10G018650.1	650-Kinase-qPCR-F	GTAACAACGAGTAGGGCACG
		650-Kinase-qPCR-R	GAGGCTGAGGAACTGATGAC
21	Soltu.DM.10G018840.1	840-nsLTP-qPCR-F	AGGCATTAACTTGTGGTCAA
		840-nsLTP-qPCR-R	TCGATCCGCGGTAGTCTTGGCTGA

Supplemental Table S5：Information of the markers used in this study

	**Name**	**Primer sequence**	**Product size (bp)**	**Tm (°C)**	**Restriction enzyme**
1	SCAR10-134	CTTGGAGACGAGTTATGAACG	530	55	
		AAAGAGAGAAAGGGGTGGC			
2	SCAR24S1	TGAGGTCAAATAGAACATAATGATA	620	52	
		ATCTAATAACTGTATGTTTATATAGATATCAA			
3	13F-SCAR	CGAACCATCTCCTATTCCG	330	55	
		CGTTGAAGAAGACGACAAAG			
4	PA28-dCAPS	ATTACCAACGTCAACCTCTGA	500	55	AflIII
		GAGTCTGGAACCTAAATAATGAAA			
5	336-CAPS	TTGAACACGTAGTCCACTGCCACT	900	61	SspI
		TTATGCGCTACTCGCGGGTAAACT			
6	1137-CAPS	ATGGTTGACACCCTCTCCATGTGT	908	60	PvuII
		AGTGGCCAGTAAATGGCTCCCATA			
7	1137-CAPSIII	GGCGTTCATATGGAACACTAGGTC	928	61	BsaBI
		GATCCTACCAGTACACTGTGGTTG			
8	1137-CAPSIV	CAAACCCAAGACCTCCTTATC	727	60	FokI
		ACCAACAAACTCCAGTCTTC			

	Name	Primer sequence	Product size (bp)	Tm (°C)	Restriction enzyme
9	1137-CAPSⅥ	GTGTCTCCTGTGAGAGATTTG	806	60	AflII
		TATAGATTGGAATGCGAGAACC			
10	1874-CAPS	GGCAAAGAGCAGTTGGTTCCCATT	833	60	BstYI
		TCGTAATTGGTGGCATCACGAGGA			
11	1137CAPSⅡ	AGTTCTCATGGTGGGCCTTTCAGA	773	61	AflII
		TATGTAGCCTGAGCTGTGTTGCCT			
12	8-CAPS	TCAAGGTCGACGCAACTCAAGTCT	800	60	SpeI
		ACCAAGCTAGCTGGAACTTTCGGA			
13	Eyd4	GGTGAACTCTCTCAATTC	574	55	BsaXI
		CCTAATATAGCTGATTCCTTGTTCC			
14	Eyd15	GCTCCTTGAGTATTGTCCTATCAGC	632	60	BsaXI
		CAGTCCACCGTACATGAAGCTATAC			
15	Eyd17	GTGATGTACGCGAACTTTAC	586	60	BsaXI
		CAAGACATCTCATTCGTTGG			
16	Eyd41	CGAGTAGTCACATTGAAAG	485	55	BsaXI
		CTAATAGCATGTGACCTAGTATGC			
17	Eyd42	GACATCTTGTCAATCAACACTTCAC	771	55	BsaXI
		CAACACATGTTGACCATGTAAGGG			
18	Eyd49	GTACACCCCTACACAATAGAGTG	706	55	BsaXI
		ACCTCTTACATCTCCTCACTTGGG			
19	LRo82	TAGGTTGTTAATATGTGCCCAAAA	172	55	
		AAAAGAAAACTTGAGCGCATCG			
